# Self-Triggered Consensus of Vehicle Platoon System With Time-Varying Topology

**DOI:** 10.3389/fnbot.2020.00053

**Published:** 2020-10-14

**Authors:** Weiping Wang, Chunyang Wang, Yongzhen Guo, Xiong Luo, Yang Gao

**Affiliations:** ^1^School of Computer and Communication Engineering, University of Science and Technology Beijing, Beijing, China; ^2^Beijing Key Laboratory of Knowledge Engineering for Materials Science, Beijing, China; ^3^Institute of Artificial Intelligence, University of Science and Technology Beijing, Beijing, China; ^4^Shunde Graduate School, Beijing University of Science and Technology, Guangzhou, China; ^5^Industrial Control System Evaluation and Certification Department of China Software Testing Center, Beijing, China; ^6^School of Automation, Beijing Institute of Technology, Beijing, China; ^7^China Information Technology Security Evaluation Center, Beijing, China

**Keywords:** consense, event-triggered, self-triggered, distributed control, time-varying topology

## Abstract

This paper focuses on the consensus problem of a vehicle platoon system with time-varying topology via self-triggered control. Unlike traditional control methods, a more secure event-triggered controller considering the safe distance was designed for the vehicle platoon system. Then, a Lyapunov function was designed to prove the stability of the platoon system. Furthermore, based on the new event-triggered function, a more energy efficient self-triggered control strategy was designed by using the Taylor formula. The new self-triggered control strategy can directly calculate the next trigger according to the state information of the last trigger. It avoids continuous calculation and measurement of vehicles. Finally, the effectiveness of the proposed two self-triggered control strategies were verified by numerical simulation experiments.

## 1. Introduction

In recent years, multi-agent systems have been widely applied in intelligent transportation (Hee Lee et al., 2013; Vilarinho et al., 2016). As an important part of the intelligent transportation system, the self-driving vehicle platoon system has a wide range of applications in improving road utilization, enhancing safety and reliability, and alleviating traffic congestion.

The formation of control is an important issue for the vehicle platoon system. It refers to the control problem that a group of intelligent vehicles can interact with each other to maintain a predetermined geometric formation during the movement of a specific target or direction. In general, this mutual interaction between intelligent vehicles can be divided into fixed and time-varying topology. Most of the current research is mainly focused on a fixed topology (Peters et al., 2016; Viegas et al., 2018). However, in the actual driving process, the vehicle platoon system often has to face various complex terrain and traffic conditions. Formations do not stay the same all the time. The vehicle platoon system requires a change of formation. Therefore, it is necessary to study the time-varying topology of vehicle platoon system. At present, there are few research studies on vehicle platoon systems with time-varying topology. More research is focused on multi-agent systems (Munz et al., 2011; Saboori and Khorasani, 2014). For example, we found that we can design more reasonable and effective control strategies by analyzing the derivatives of time-varying topological variables (Wang et al., 2018). It is thus more practical to study the time-varying topology of the vehicle platoon system than fixed topology.

Recently, the formation consistency of the vehicle platoon system has been widely considered. It has been applied to deal with consistency of formation control problems (Ren, 2007; Stojković and Katić, 2017; Wang et al., 2017; Li et al., 2018). Bela Lantos and Gyorgy Max achieved the formation consistency of unmanned ground vehicles by using a two-trajectory non-linear dynamic model (Lantos and Max, 2016). Peters et al. (2016) designed a way by which each follower tracks its immediate predecessor to achieve vehicle formation consistency.

Nevertheless, in the traditional vehicle platoon system consistency study, it is assumed that the vehicle platoon system has sufficient computing resources and energy supply (Fax and Murray, 2004; Lafferriere et al., 2005). The vehicle platoon system can thus carry on a continuous information exchange and a continuous control. However, such assumption is unreasonable. More often than not, the power supply and communication bandwidth of a vehicle platoon system are limited. Recently, it has been found that event-triggered control can coordinate resources among intelligent vehicles. Many scholars are thus interested in event-triggered control. As an aperiodic control mode, event-triggered control can update the controller only when needed. That is, the controller of the intelligent vehicle takes an effect when the measurement error of the vehicle platoon system exceeds a certain threshold.

Since event-triggered control can reduce the energy loss to a certain extent, many scholars apply it to consistency research (Wei et al., 2017). The author in Chu et al. (2019) proposed an unified event-triggered and distributed observer-based controller with globally asymptotic convergence rate. The consistency of vehicle platoon system is realized by the controller. A fault-tolerant controller which considered the communication time-delay and event-triggered mechanism was designed to achieve the consistency of the vehicle platoon system (Fei et al., 2019).

However, in order to obtain the next trigger moment, we need to constantly obtain the state information of surrounding vehicles and calculate whether the trigger conditions are met in the distributed event-triggered control function. It is because of continuous communication and computation that an event-triggered control strategy cannot reduce the detection loss in essence. But the self-triggered control strategy only needs to calculate the next trigger moment based on the status information of the last trigger moment. In the self-triggered strategy, data detection is no longer required between any two triggering moments. From this perspective, the self-triggered control strategy has a better performance. Authors designed a self-triggered control strategy for the second-order multi-agent system with fixed topology to ensure the consistency of the formation system (De Persis and Frasca, 2013). As far as we know, there are few research studies made on time-varying topology under self-triggering control in vehicle platoon system, and this sparked our research.

Based on the above considerations, we studied the consistency of time-varying topology for vehicle platoon system with second-order dynamics by using distributed event-triggered control and self-triggered control strategies. The contributions of our work are three-fold:

1. A distributed event-triggered control function considering the safe distance between vehicles was designed, and this event-triggered control is more energy efficient than the continuous control in Fax and Murray (2004) and Lafferriere et al. (2005).
2. Based on the Lyapunov stability analysis method, the distributed event-triggered control function under time-varying leader and time-varying topology was given. In comparison with the fixed topology in Du et al. (2017), the research of time-varying topology is more practical. Moreover, the research on time-varying leader is of more practical significance.
3. According to (1) and (2), two distributed self-triggered control strategies were designed. In Zhang et al. (2016), Dolk et al. (2017), Wei et al. (2017), Wen et al. (2018), Chu et al. (2019), and Li Z. et al. (2019), an event-triggered control strategy was designed. Compared with these, the self-triggered control strategy further reduces the continuous detection of adjacent vehicles. Additionally, the distributed self-triggered controller is more general and practical than some existing control methods.

The rest of this paper was organized as follows. Preliminaries and the problem formulation are given in section 2. The event-triggered control and self-triggered control of vehicle platoon system with time-varying topology are studied in section 3. Two numerical simulation experiments are presented in section 4. Lastly, conclusions are drawn in section 5.

## 2. Preliminaries and Problem Formulation

### 2.1. Graph Theory

Consider the consensus issue of multi-agent systems with time-varying topology; a communication graph is used to describe the communication topology of these agents. An undirected graph G=(V,E,A) consists of a finite node set V={1,2,⋯,N}, an edge set E, where E⊆V×V, and an adjacency matrix $A=\left[a_{ij}\right]\in {\mathbb{R}}^{N\times N}$. If (j,i)∈E, *a*_*ij*_ = 1, and *a*_*ij*_ = 0 otherwise. The neighbor set of vehicle *i* is defined as $N_{i}≜\left\{j\in V|\left(j,i\right)\in E,j\neq i\right\}$. The Laplacian matrix of the graph G is defined as $L=\left[l_{ij}\right]\in {\mathbb{R}}^{N\times N}$, where $l_{ii}=\underset{j\neq i}{\sum }a_{ij}$ and *l*_*ij*_ = −*a*_*ij*_, where *i* ≠ *j*. Moreover, we assume that there are no self-cycles, that is *a*_*ii*_ = 0 for any $i\in \bar{N}$. The degree matrices $D=\mathrm{diag}\left\{d_{1},\cdots \;,d_{N}\right\}$ are diagonal matrices, whose diagonal elements are given by $d_{i}=\overset{N}{\underset{j=1}{\sum }}a_{ij}$. The Laplacian matrix associated to G is defined as L=D-A. The set of all neighbors of node *i* is denoted by $N_{i}=\left\{j\in V:\left(j,i\right)\in E\right\}$. The matrix *B* = diag{*b*_1_, *b*_2_, ⋯ , *b*_*N*_}, where *b*_*i*_ is called the adjacency coefficient between the following vehicle *i* and the head vehicle. If the following vehicle *i* is adjacent to the head vehicle *b*_*i*_ = 1, otherwise *b*_*i*_ = 0. In this paper, we define the time interval constant *h*_*ij*_ > 0 to control the safe distance between vehicles *i* and *j*. At the same time, we define *h*_*i*_ > 0 to control the safe distance between vehicle *i* and the leader vehicle.

### 2.2. Definitions and Lemmas

**ASSUMPTION 2.1**. *It is assumed that no topology changes happen during the trigger interval*.

**ASSUMPTION 2.2**. *It is assumed that the communication between vehicles is good, that is, there will be no communication delay and other uncertain factors*.

**ASSUMPTION 2.3**. *Suppose that at least one spanning tree exists in G and the node corresponding to the header is the root of the tree. The existence of the spanning tree ensures that each following vehicle can obtain the status information from the leader*.

**LEMMA 2.1**. $2x^{T}y\leq ax^{T}x+\frac{1}{a}y^{T}y$, *where *a* > 0, and the vectors *x* and *y* can be any value*.

**LEMMA 2.2**. *Satur and Kharchenko (2020) suppose the matrix *A* is a *n* × *n* real symmetric matrix, *Y* is an n-dimensional real vector, and λ_max_(*A*) ≥ λ_*i*_(*A*) ≥ λ_min_(*A*)(*i* = 1, 2, ..., *N*). One has*

(1) $$\lambda_{\max}(A)\langle Y,Y\rangle \geq \langle AY,Y\rangle \geq \lambda_{\min}(A)\langle Y,Y\rangle .$$

**LEMMA 2.3**. *Li W. et al. (2019) assuming that the function *f* satisfies Lipschitz condition, there is a non-negative constant *l* ≥ 0 that satisfies ||*f*(*t, x*_*i*_) − *f*(*t, x*_0_)|| ≤ *l*||*x*_*i*_ − *x*_0_||, or there are non-negative constants *l*_*x*_ ≥ 0, *l*_*v*_ ≥ 0 satisfies*

(2) $$\begin{array}{r} \begin{array}{c} ||f\left(t,x_{i},v_{i}\right)-f\left(t,x_{0},v_{0}\right)||\leq l_{x}||x_{i}-x_{0}||+l_{v}||v_{i}-v_{0}||. \end{array} \end{array}$$

### 2.3. Problem Formulation

**Figure 1:**
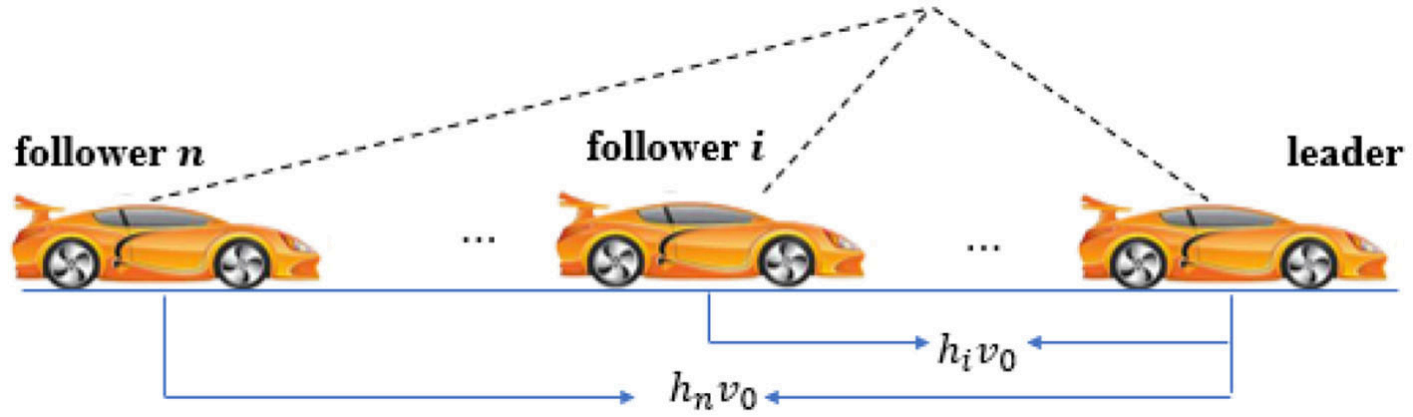
Platoon of vehicles.

An auto-driving vehicle formation system consisted of *n* smart cars (see Figure 1) is considered in this paper. Between the vehicles, status information can be transmitted according to certain regulations. The *h*_*i*_*v*_0_ in Figure 1 is the distance between the *i* th vehicle and the leader.

In this paper, the dynamics of the leader vehicle is described as

(3) $$\{\begin{array}{l} {\dot{x}}_{0}(t)=v_{0}(t),\\ {\dot{v}}_{0}(t)=f(t,x_{0},v_{0}), \end{array}$$

where $x_{0}\left(t\right),v_{0}\left(t\right)\in {\text{R}}^{m}$ express the displacement vector and the velocity vector of the leader vehicle, and *f*(*t, x*_0_, *v*_0_) is the control input of the leader vehicle. When *f* = 0, the velocity of leader vehicle is constant, when *f* ≠ 0 the velocity of the leader vehicle is changing.

The dynamic equation of the *i* th follower intelligent vehicle is described as

(4) $$\{\begin{array}{l} {\dot{x}}_{i}(t)=v_{i}(t),\\ {\dot{v}}_{i}(t)=f(t,x_{i},v_{i})-f(t,x_{0},v_{0})+u_{i}(t), \end{array}$$

where $x_{i}\left(t\right),v_{i}\left(t\right)\in {\text{R}}^{m}$ express the displacement vector and the speed vector of the follower intelligent vehicle *i* respectively, and *f*(*t, x*_*i*_, *v*_*i*_) − *f*(*t, x*_0_, *v*_0_) + *u*_*i*_(*t*) is the control input of the *i* th follower intelligent vehicle.

**REMARK 1**. *The function *f*(*t, x, v*) is an acceleration function known to all vehicles, and it satisfies LEMMA 2.3*.

## 3. Main Result

### 3.1. Distributed Event-Triggered Control of Vehicle Platoon System With Time-Varying Topology

In order to reduce the sensor data acquisition and the energy consumption of frequent communication between vehicles, a distributed event-triggered controller is designed in this section. In the distributed event-triggered controller, each of the following vehicle has different trigger function, and its controller update is asynchronous. When the trigger condition is satisfied, the controller of the *i* th follower vehicle is updated at ${t_{k}}^{i}\left(k=0,1,2\cdots \;\right)$. It is satisfied $u_{i}\left(t\right)=u_{i}\left({t_{k}}^{i}\right)$, and $\overset{∙}{u_{i}}\left(t\right)=0$, $\forall t\in \left[{t_{k}}^{i},{t_{k}}^{i+1}\right)$. Since the topology is time-varying, the graph G can be treated as G(t). Accordingly, A, L, D, and B become A(t), L(t), D(t), and B(t). In this case, Assumption 1 and Assumption 2 still hold.

#### 3.1.1. The Leader Vehicle Speed Is Constant

In this section, the leader-follower consistency problem in the case of time-varying topology, which is based on the fact that the leader vehicle speed is constant, is studied, i.e., *f*(*t, x*_0_, *v*_0_) = 0.

To make the system consistent, we set the controller of *i* th follower vehicle:

(5) $$\begin{array}{l} u_{i}(t)=-k[\sum_{j\in N_{i}(t_{k}{}^{i})}a_{ij}(t_{k}{}^{i})(x_{i}(t_{k}{}^{i})-x_{j}(t_{k}{}^{i})-h_{ij}v_{0})\\ \;+b_{i}(t_{k}{}^{i})(x_{i}(t_{k}{}^{i})-x_{0}(t_{k}{}^{i})-h_{i}v_{0})]\\ \;-kr[\sum_{j\in N_{i}(t_{k}{}^{i})}a_{ij}(t_{k}{}^{i})(v_{i}(t_{k}{}^{i})-v_{j}(t_{k}{}^{i}))+b_{i}(t_{k}{}^{i})(v_{i}(t_{k}{}^{i})\\ \;-v_{0}(t_{k}{}^{i}))],\;t\in [t_{k}{}^{i},t_{k}{}^{i+1}), \end{array}$$

where *k* and *r* are control gains, and $N_{i}\left({t_{k}}^{i}\right)$ represents the set of neighbors of the *i* th follower vehicle at ${t_{k}}^{i}$.

In order to describe the displacement and speed tracking between the following vehicle *i* and the leader vehicle, we defined the displacement error ε_*i*_ and the velocity error η_*i*_ as follows:

(6) $$\begin{array}{l} \varepsilon_{i}(t)=x_{i}(t)-x_{0}(t)-h_{i}v_{0},\\ \eta_{i}(t)=v_{i}(t)-v_{0}(t). \end{array}$$

The measurement error $e_{i}^{x}\left(t\right)$ and $e_{i}^{v}\left(t\right)$ are designed to represent the displacement and velocity differences between the triggering and the measuring moments of the *i* th follower vehicle. We have then

(7) $$\begin{array}{l} e_{i}^{x}(t)=\varepsilon_{i}(t_{k}^{i})-\varepsilon_{i}(t),\\ e_{i}^{v}(t)=\eta_{i}(t_{k}^{i})-\eta_{i}(t). \end{array}$$

So the controller of the following intelligent vehicle becomes

(8) $$\begin{array}{l} u_{i}(t)=-k[\sum_{j\in N_{i}(t)}a_{ij}(t)(\varepsilon_{i}(t)-\varepsilon_{j}(t))+b_{i}(t)\varepsilon_{i}(t)]\\ \;-kr[\sum_{j\in N_{i}(t)}a_{ij}(t)(\eta_{i}(t)-\eta_{j}(t))+b_{i}(t)\eta_{i}(t)]\\ \;-k[\sum_{j\in N_{i}(t)}a_{ij}(t)(e_{i}{}^{x}(t)-e_{j}{}^{x}(t))+b_{i}(t)e_{i}{}^{x}(t)]\\ \;-kr[\sum_{j\in N_{i}(t)}a_{ij}(t)(e_{i}{}^{v}(t)-e_{j}{}^{v}(t))+b_{i}(t)e_{i}{}^{v}(t)]. \end{array}$$

The states and the measurement errors of intelligent vehicle are written in the vector form:

(9) $$\begin{array}{l} \varepsilon (t)=\text{col}\left(\varepsilon_{1}(t),\varepsilon_{2}(t),\ldots ,\varepsilon_{N}(t)\right),\\ \eta (t)=\text{col}\left(\eta_{1}(t),\eta_{2}(t),\ldots ,\eta_{3}(t)\right),\\ e^{x}(t)=\text{col}\left(e_{1}^{x}(t),e_{2}^{x}(t),\ldots ,e_{N}^{x}(t)\right),\\ e^{v}(t)=\text{col}\left(e_{1}^{v}(t),e_{2}^{v}(t),\ldots ,e_{N}^{v}(t)\right). \end{array}$$

According to (9), we have

(10) $$\{\begin{array}{l} \dot{\varepsilon }(t)=\eta (t),\\ \dot{\eta }(t)=-k(L(t)+B(t))⊗I_{m}\varepsilon (t)-rk(L(t)+B(t))\\ \;⊗I_{m}\eta (t)-k(L(t)+B(t))⊗I_{m}e^{x}(t)\\ \;-rk(L(t)+B(t))⊗I_{m}e^{v}(t). \end{array}$$

**Theorem 3.1**. *Consider a fleet of *N* + 1 vehicles, where the dynamic equations of the head vehicle and the follower vehicle are (3) and (4). If the following conditions are met under the controller (8), then*

(1) The proposed event triggering function satisfies

(11) $$\zeta \left({\left\|\varepsilon_{i}(t)\right\|}^{2}+{\left\|\eta_{i}(t)\right\|}^{2}\right)\leq M\left({\left\|e_{i}^{x}(t)\right\|}^{2}+{\left\|e_{i}^{v}(t)\right\|}^{2}\right),$$

*where $M=\frac{ka\lambda_{\min}\left(H\left(t\right)\right)}{2}$, *H*(*t*) = *L*(*t*) + *B*(*t*) and ζ will be indicated below. When this condition is met, the controller automatically updates, that is, the trigger time is reached*.

*(2) The minimum eigenvalue of (*L*(*t*) + *B*(*t*))⊗*I*_*m*_ is greater than zero, which is greater than an arbitrarily small normal number δ*.

(12) $$\lambda_{\min}\left((L(t)+B(t))⊗I_{m}\right)\geq \delta >0.$$

(3) The differential coefficient of (*L*(*t*) + *B*(*t*))⊗*I*_*m*_ exists, and the maximum eigenvalue of its derivative is greater than zero; for any small positive number, σ is satisfied

(13) $$\lambda_{\max}\left(d\left((L(t)+B(t))⊗I_{m}\right)/\text{d}t\right)\geq \sigma >0,$$

(4) The relation between η(*t*) and *e*^*x*^(*t*), *e*^*v*^(*t*) is

(14) $$\begin{array}{l} -<kH(t)⊗I_{m}e^{x}(t),\eta (t)>\;\leq -\frac{ka\lambda_{\min}(H(t))}{2}||e^{x}(t)||\\ \text{ +}\frac{k\lambda_{\min}(H(t))}{2a}||\eta (t)||,\\ -<kH(t)⊗I_{m}e^{v}(t),\eta (t)>\;\leq -\frac{ka\lambda_{\min}(H(t))}{2}||e^{v}(t)||\\ \text{ +}\frac{k\lambda_{\min}(H(t))}{2a}||\eta (t)||, \end{array}$$

where $\zeta =\max\left(\frac{k\psi }{2},\left(\frac{1}{a}-1\right)k\lambda_{\min}\left(H\left(t\right)\right)\right),0<a<1,$ ψ = λ_max_(d((*L*(*t*)+*B*(*t*))⊗*I*_*m*_)/d*t*), then all the vehicle reach the same state, and the existence of the safe distance *h*_*ij*_*v*_0_ avoid a collision. Hence, the problem of intelligent vehicle formation is solved, i.e., for *i* = 1, 2, …, *N*, we have

$$\begin{array}{l} \underset{t\to \infty }{\lim}\left\|\varepsilon (t)\right\|=0,\\ \underset{t\to \infty }{\lim}\left\|\eta (t)\right\|=0. \end{array}$$

**PROOF**. Based on system (10), we can construct the common Lyapunov function candidate

(15) $$\begin{array}{l} V(t)\text{=}\int_{0}^{1}<kH(t)⊗I_{m}\omega \varepsilon (t),\varepsilon (t)>d\omega \\ \;+\frac{1}{2}<\eta (t),\eta (t)>, \end{array}$$

where *H*(*t*) = *L*(*t*)+*B*(*t*).

Firstly, we prove the positivity of *V*(*t*)

(16) $$\begin{array}{l} V\geq \frac{1}{2}k\lambda_{\min}\left(H(t)⊗I_{m}\right){\left\|\varepsilon (t)\right\|}^{2}+\frac{1}{2}\langle \eta (t),\eta (t)\rangle \\ \text{ =}\frac{1}{2}k\lambda_{\min}\left(H(t)⊗I_{m}\right){\left\|\varepsilon (t)\right\|}^{2}+\frac{1}{2}{\left\|\eta (t)\right\|}^{2}\\ \;\geq \frac{1}{2}\min\left\{k\zeta ,1\right\}\left({\left\|\varepsilon (t)\right\|}^{2}+{\left\|\eta (t)\right\|}^{2}\right). \end{array}$$

It can be seen that the Lyapunov function (15) selected is positively definite.

The time derivative of (15) can be expressed as

(17) $$\begin{array}{l} \frac{\text{d}V}{\text{d}t}=\frac{\text{d}}{\text{d}t}\int_{0}^{1}<kH(t)⊗I_{m}\omega \varepsilon (t),\varepsilon (t)>d\omega \;\\ \;-<kH(t)⊗I_{m}\varepsilon (t),\eta (t)>\\ \;-<kH(t)⊗I_{m}\eta (t),\eta (t)>\\ \;-<kH(t)⊗I_{m}e^{x}(t),\eta (t)>\\ \;-<kH(t)⊗I_{m}e^{v}(t),\eta (t)>. \end{array}$$

Taking out the first term, we have

(18) $$\begin{array}{l} \frac{d}{\text{d}t}\int_{0}^{1}<kH(t)⊗I_{m}\omega \varepsilon (t),\varepsilon (t)>d\omega \\ \;=\int_{0}^{1}<kH(t)⊗I_{m}\omega \eta (t),\varepsilon (t)>d\omega \\ \text{ +}\int_{0}^{1}<kH(t)⊗I_{m}\omega \varepsilon (t),\eta (t)>d\omega \\ \text{ +}\int_{0}^{1}<k\frac{\text{d}(H(t))}{\text{d}t}⊗I_{m}\omega \varepsilon (t),\varepsilon (t)>d\omega \\ \;=<kH(t)⊗I_{m}\varepsilon (t),\eta (t)>\\ \;+\int_{0}^{1}<k\frac{\text{d}(H(t))}{\text{d}t}⊗I_{m}\omega \varepsilon (t),\varepsilon (t)>d\omega . \end{array}$$

By (18), we get

(19) $$\begin{array}{l} \frac{\text{d}V}{\text{d}t}=\int_{0}^{1}<k\frac{\text{d}(\text{H}(\text{t})⊗{\text{I}}_{\text{m}})}{\text{d}t}\omega \varepsilon (t),\varepsilon (t)>d\omega -<kH(t)\\ \;⊗I_{m}\eta (t),\eta (t)>-<kH(t)⊗I_{m}e^{x}(t),\eta (t)>\\ \;-<kH(t)⊗I_{m}e^{v}(t),\eta (t)>. \end{array}$$

Take (19) into consideration, we have

(20) $$\begin{array}{l} \frac{\text{d}V}{\text{d}t}=\int_{0}^{1}<k\frac{\text{d}(H(t)⊗I_{m})}{\text{d}t}\omega \varepsilon (t),\varepsilon (t)>d\omega \\ \;-<kH(t)⊗I_{m}\eta (t),\eta (t)>\\ \;-\frac{ka\lambda_{\min}(H(t))}{2}||e^{x}(t)|{|}^{2}\text{+}\frac{k\lambda_{\min}(H(t))}{2a}||\eta (t)|{|}^{2}\\ \;-\frac{ka\lambda_{\min}(H(t))}{2}||e^{v}(t)|{|}^{2}\text{+}\frac{k\lambda_{\min}(H(t))}{2a}||\eta (t)|{|}^{2}\\ \;\leq (\frac{k\psi }{2}||\varepsilon (t)|{|}^{2}+(\frac{1}{a}-1)k\lambda_{\min}(H(t))||\eta (t)|{|}^{2})\\ \;-(\frac{ka\lambda_{\min}(H(t))}{2}(||e^{x}(t)|{|}^{2}+||e^{v}(t)|{|}^{2}))\\ \;\leq \varsigma (||\varepsilon (t)|{|}^{2}+||\eta (t)|{|}^{2})\\ \;-(\frac{ka\lambda_{\min}(H(t))}{2}(||e^{x}(t)|{|}^{2}+||e^{v}(t)|{|}^{2})), \end{array}$$

where $\zeta =\text{max}\left(\frac{k\psi }{2},\left(\frac{1}{a}-1\right)k\lambda_{\min}\left(H\left(t\right)\right)\right)$, ψ = λ_max_(d((*L*(*t*)+*B*(*t*))⊗*I*_*m*_)/d*t*). According to the trigger condition (11), the derivative of the Lyapunov function is less or equal to 0, so the stability is proved.

#### 3.1.2. The Speed of the Leading Vehicle Is Time Varying

In the actual situation, the speed of the leading vehicle cannot be fixed, most of them are time varying. Therefore, in this section, we study the consistency of leader followers in the case of time-varying topology based on the fact that the speed of the leader vehicle is time varying, i.e., *f*(*t, x*_0_, *v*_0_)≠0. In the meantime, suppose $f\left(t,\varepsilon_{i}\left(t\right),\eta_{i}\left(t\right)\right)=f\left({t_{k}}^{i},x_{i}\left({t_{k}}^{i}\right),v_{i}\left({t_{k}}^{i}\right)\right)-f\left(t,x_{0}\left({t_{k}}^{i}\right),v_{0}\left({t_{k}}^{i}\right)\right)$.

To make the system consistent, we set the *i* th follower vehicle's controller as

(21) $$\begin{array}{l} u_{i}(t)=-k[\sum_{j\in N_{i}(t_{k}{}^{i})}a_{ij}(t_{k}{}^{i})(x_{i}(t_{k}{}^{i})-x_{j}(t_{k}{}^{i})-h_{ij}v_{0})\\ \;+b_{i}(t)(x_{i}(t_{k}{}^{i})-x_{0}(t_{k}{}^{i})-h_{i}v_{0})]\\ \;-kr[\sum_{j\in N_{i}(t_{k}{}^{i})}a_{ij}(t_{k}{}^{i})(v_{i}(t_{k}{}^{i})-v_{j}(t_{k}{}^{i}))+b_{i}(t_{k}{}^{i})(v_{i}(t)\\ \;-v_{0}(t_{k}{}^{i}))],\;t\in [t_{k}{}^{i},t_{k}{}^{i+1}). \end{array}$$

Similar to (6)–(9), we can format the system (4) as follows

(22) $$\begin{array}{l} \dot{\varepsilon }(t)=\eta (t),\\ \dot{\eta }(t)=f(t,\varepsilon (t),\eta (t),e^{x}(t),e^{v}(t))-k(L(t)+B(t))⊗I_{m}\varepsilon (t)\\ \;-rk(L(t)+B(t))⊗I_{m}\eta (t)-k(L(t)+B(t))⊗I_{m}e^{x}(t)\\ \;-rk(L(t)+B(t))⊗I_{m}e^{v}(t) \end{array}$$

**Theorem 3.2**. *Consider a fleet of *N*+1 vehicles, where the dynamic equations of the head vehicle and the follower vehicle are (3) and (4) respectively. If the following conditions are met under the controller (21), then*

*(1) The designed event triggering function satisfies the following conditions*.

(23) $$\begin{array}{l} \zeta (||\varepsilon_{i}(t)|{|}^{2}+||\eta_{i}(t)|{|}^{2})\leq (\frac{ka\lambda_{\min}(H(t))}{2}-\frac{l}{2a_{1}})\\ \;\times (||e_{i}{}^{x}(t)|{|}^{2}+||e_{i}{}^{v}(t)|{|}^{2})), \end{array}$$

*where *H*(*t*) = *L*(*t*)+*B*(*t*) and ζ will be indicated below. When this condition is met, the controller automatically updates, that is, the trigger time is reached*.

(2) The minimum eigenvalue of (*L*(*t*)+*B*(*t*))⊗*I*_*m*_ is greater than zero. There is thus a small positive number δ satisfying

(24) $$\lambda_{\min}\left((L(t)+B(t))⊗I_{m}\right)\geq \delta >0.$$

(3) The differential coefficient of (*L*(*t*)+*B*(*t*))⊗*I*_*m*_ exists, and the maximum eigenvalue of its derivative is greater than zero, so there exists a small positive number σ satisfying

(25) $$\lambda_{\max}\left(\text{d}\left((L(t)+B(t))⊗I_{m}\right)/\text{d}t\right)\geq \sigma >0.$$

(4) The relation between η(*t*) and *e*^*x*^(*t*), *e*^*v*^(*t*) is

(26) $$\begin{array}{l} -<kH(t)⊗I_{m}e^{x}(t),\eta (t)>\;\leq -\frac{ka\lambda_{\min}(H(t))}{2}||e^{x}(t)||\\ \text{ +}\frac{k\lambda_{\min}(H(t))}{2a}||\eta (t)||\\ -<kH(t)⊗I_{m}e^{v}(t),\eta (t)>\;\leq -\frac{ka\lambda_{\min}(H(t))}{2}||e^{v}(t)||\\ \text{ +}\frac{k\lambda_{\min}(H(t))}{2a}||\eta (t)||. \end{array}$$

(5)

(27) $$\frac{ka\lambda_{\min}(H(t))}{2}-\frac{l}{2a_{1}}>0,$$

where $\zeta =\max\left(\frac{k\psi }{2}+\frac{l}{2a_{1}},\frac{3a_{1}l}{2}+l+\frac{k\lambda_{\min}\left(H\left(t\right)\right)}{a}\right)$, ψ = λ_max_(d((*L*(*t*)+*B*(*t*))⊗*I*_*m*_)/d*t*), 0 < *a*, 0 < *a*_1_, 0 < *k*, then all the vehicles reach the same state, and at the same time the existence of safe distance *h*_*ij*_*v*_0_ avoid a collision. The problem of intelligent vehicle formation has been solved, i.e., for *i* = 1, 2, …, *N*, we have

$$\begin{array}{l} \underset{t\to \infty }{\lim}\left\|\varepsilon (t)\right\|=0,\\ \underset{t\to \infty }{\lim}\left\|\eta (t)\right\|=0. \end{array}$$

**PROOF**. Based on system (22), we can construct the Lyapunov function candidate

(28) $$V(t)\text{=}\int_{0}^{1}<kH(t)⊗I_{m}\omega \varepsilon (t),\varepsilon (t)>d\omega +\frac{1}{2}<\eta (t),\eta (t)>,$$

where *H*(*t*) = *L*(*t*)+*B*(*t*).

It can be seen that the (28) selected is positively definite.

The time derivative of (28) can be expressed as

(29) $$\begin{array}{l} \frac{\text{d}V}{\text{d}t}=\frac{\text{d}}{\text{d}t}\int_{0}^{1}<kH(t)⊗I_{m}\omega \varepsilon (t),\varepsilon (t)>d\omega \\ \;+<\eta (t),f(t,\varepsilon (t),\eta (t),e^{x}(t),e^{v}(t))>\\ \;-<kH(t)⊗I_{m}\varepsilon (t),\eta (t)>-<kH(t)⊗I_{m}\eta (t),\eta (t)>\\ \;-<kH(t)⊗I_{m}e^{x}(t),\eta (t)>-<kH(t)⊗I_{m}e^{v}(t),\eta (t)>. \end{array}$$

Taking out of the first term, we have

(30) $$\begin{array}{l} \frac{d}{\text{d}t}\int_{0}^{1}<kH(t)⊗I_{m}\omega \varepsilon (t),\varepsilon (t)>d\omega \\ =\int_{0}^{1}<kH(t)⊗I_{m}\omega \eta (t),\varepsilon (t)>d\omega \\ \text{+}\int_{0}^{1}<kH(t)⊗I_{m}\omega \varepsilon (t),\eta (t)>d\omega \\ \text{+}\int_{0}^{1}<k\frac{\text{d}(H(t))}{\text{d}t}⊗I_{m}\omega \varepsilon (t),\varepsilon (t)>d\omega \\ =<kH(t)⊗I_{m}\varepsilon (t),\eta (t)>\\ +\int_{0}^{1}<k\frac{\text{d}(H(t)⊗I_{m})}{\text{d}t}\omega \varepsilon (t),\varepsilon (t)>d\omega . \end{array}$$

Using Lemma 2 to enlarge the second item in (29), we yield

(31) $$\begin{array}{l} <\eta (t),f(t,\varepsilon (t),\eta (t),e^{x}(t),e^{v}(t))>\\ \;\leq \left\|\eta (t)\right\|l(\left\|\varepsilon (t)\right\|+\left\|\eta (t)\right\|\\ \;+\left\|e^{x}(t)\right\|+\left\|e^{v}(t)\right\|). \end{array}$$

Considering (30) and (31), we get

(32) $$\begin{array}{l} \frac{\text{d}V}{\text{d}t}\leq \int_{0}^{1}<k\frac{\text{d}(H(t)⊗I_{m})}{dt}\omega \varepsilon (t),\varepsilon (t)>d\omega \\ \text{ +}\left\|\eta (t)\right\|l(\left\|\varepsilon (t)\right\|+\left\|\eta (t)\right\|+\left\|e^{x}(t)\right\|+\left\|e^{v}(t)\right\|)\\ \;-<kH(t)⊗I_{m}\eta (t),\eta (t)>\\ \;-<kH(t)⊗I_{m}e^{x}(t),\eta (t)>-<kH(t)⊗I_{m}e^{v}(t),\eta (t)>. \end{array}$$

By using Lemma 3, the above items are amplified, and then

(33) $$\begin{array}{l} \left\|\eta (t)\right\|l(\left\|\varepsilon (t)\right\|+\left\|\eta (t)\right\|+\left\|e^{x}(t)\right\|+\left\|e^{v}(t)\right\|)\\ \text{=}l(\left\|\eta (t)\right\|\left\|\varepsilon (t)\right\|+\left\|\eta (t)\right\|\left\|\eta (t)\right\|\\ +\left\|\eta (t)\right\|\left\|e^{x}(t)\right\|+\left\|\eta (t)\right\|\left\|e^{v}(t)\right\|), \end{array}$$

(34) $$\begin{array}{l} \left\|\eta (t)\right\|\left\|\varepsilon (t)\right\|\leq \frac{a_{1}}{2}{\left\|\eta (t)\right\|}^{2}+\frac{1}{2a_{1}}{\left\|\varepsilon (t)\right\|}^{2},\\ \left\|\eta (t)\right\|\left\|e^{x}(t)\right\|\leq \frac{a_{1}}{2}{\left\|\eta (t)\right\|}^{2}+\frac{1}{2a_{1}}{\left\|e^{x}(t)\right\|}^{2},\\ \left\|\eta (t)\right\|\left\|e^{v}(t)\right\|\leq \frac{a_{1}}{2}{\left\|\eta (t)\right\|}^{2}+\frac{1}{2a_{1}}{\left\|e^{v}(t)\right\|}^{2}, \end{array}$$

where *a*_1_>0.

Considering (34), we have

(35) $$\begin{array}{l} \frac{\text{d}V}{\text{d}t}\leq \frac{k\psi }{2}{\left\|\varepsilon (t)\right\|}^{2}+(\frac{a_{1}l}{2}+l){\left\|\eta (t)\right\|}^{2}\\ \;+\frac{l}{2a_{1}}{\left\|\varepsilon (t)\right\|}^{2}+\frac{a_{1}l}{2}{\left\|\eta (t)\right\|}^{2}+\frac{l}{2a_{1}}{\left\|e^{x}(t)\right\|}^{2}\\ \;+\frac{a_{1}l}{2}{\left\|\eta (t)\right\|}^{2}+\frac{l}{2a_{1}}{\left\|e^{v}(t)\right\|}^{2}-\frac{ka\lambda_{\min}(H(t))}{2}||e^{x}(t)||\\ \text{ +}\frac{k\lambda_{\min}(H(t))}{2a}||\eta (t)||\\ \;-\frac{ka\lambda_{\min}(H(t))}{2}||e^{v}(t)||\text{+}\frac{k\lambda_{\min}(H(t))}{2a}||\eta (t)||\\ \;\leq (\frac{k\psi }{2}+\frac{l}{2a_{1}}){\left\|\varepsilon (t)\right\|}^{2}+(\frac{3a_{1}l}{2}+l+\frac{k\lambda_{\min}(H(t))}{2a}\\ \;+\frac{k\lambda_{\min}(H(t))}{2a}){\left\|\eta (t)\right\|}^{2}\\ \;-(\frac{ka\lambda_{\min}(H(t))}{2}-\frac{l}{2a_{1}})({\left\|e^{x}(t)\right\|}^{2}+{\left\|e^{v}(t)\right\|}^{2})\\ \;\leq \varsigma ({\left\|\varepsilon (t)\right\|}^{2}+{\left\|\eta (t)\right\|}^{2})-(\frac{ka\lambda_{\min}(H(t))}{2}-\frac{l}{2a_{1}})\\ \;({\left\|e^{x}(t)\right\|}^{2}+{\left\|e^{v}(t)\right\|}^{2}). \end{array}$$

According to the trigger condition (23), the derivative of the Lyapunov function (29) is less or equal to 0, and it is constant, so the stability is proved.

### 3.2. Distributed Self-Triggered Control of Vehicle Platoon System With Time-Varying Topology

As can be seen from the distributed event-triggered control (11) and (23), the control method reduces the dependence on the global state information and the real-time state of measurement error in the trigger interval. However, it will increase the energy consumption of the sensor and microprocessor in the process of continuous measurement error detection. In order to improve this problem, we apply the self-triggered control strategy to solve the problem of intelligent vehicle formation. Under this strategy, the next trigger moment $t_{k+1}^{i}$ of the *i* th follower vehicle can obtained according to the state of the *i* th vehicle at the previous trigger time.

#### 3.2.1. The Leader Vehicle Speed Is Constant

In this part, we will transform the event-triggered control (11) into a self-triggered control strategy for the case that the vehicle speed of the leader is constant.

We know that from the previous distributed event triggering control $\left(||\varepsilon_{i}\left(t\right)|{|}^{2}+||\eta_{i}\left(t\right)|{|}^{2}\right)\leq \gamma \left(||e_{i}^{x}\left(t\right)|{|}^{2}+||e_{i}^{v}\left(t\right)|{|}^{2}\right)$, where $\gamma =\frac{ka\lambda_{\min}\left(H\left(t\right)\right)}{2\zeta }$. Using Taylor's formula, expand $\varepsilon_{i},\eta_{i},e_{i}^{x},e_{i}^{v}$ at $t_{k}^{i}$, we have

(36) $$\begin{array}{l} \varepsilon_{i}(t)=\eta_{i}(t_{k}{}^{i})(t-t_{k}{}^{i})+\varepsilon_{i}(t_{k}{}^{i}),\\ \eta_{i}(t)=\dot{\eta_{i}}(t_{k}{}^{i})(t-t_{k}{}^{i})+\eta_{i}(t_{k}{}^{i})\\ \;=(-k\sum_{j=1}^{N}\left(L_{j,\cdot }(t_{k}{}^{i})+B_{j,\cdot }(t_{k}{}^{i}))\varepsilon_{j}(t_{k}{}^{i}\right)\\ \;-kr(\sum_{j=1}^{N}\left(L_{j,\cdot }(t_{k}{}^{i})+B_{j,\cdot }(t_{k}{}^{i}\right))\eta_{j}(t_{k}{}^{i})(t-t_{k}{}^{i}))\\ \;+\eta_{i}(t_{k}{}^{i}),\\ e_{i}{}^{x}=\;\varepsilon_{i}(t_{k}{}^{i})-\varepsilon_{i}(t)=-\eta_{i}(t_{k}{}^{i})(t-t_{k}{}^{i})\\ e_{i}{}^{v}=\;\eta_{i}(t_{k}{}^{i})-\eta_{i}(t)=\dot{-\eta_{i}}(t_{k}{}^{i})(t-t_{k}{}^{i})\\ \;=-(k\sum_{j=1}^{N}\left(L_{j,\cdot }(t_{k}{}^{i})+B_{j,\cdot }(t_{k}{}^{i}))\varepsilon_{j}(t_{k}{}^{i}\right)\\ \;-kr\sum_{j=1}^{N}\left(L_{j,\cdot }(t_{k}{}^{i})+B_{j,\cdot }(t_{k}{}^{i}))\eta_{j}(t_{k}{}^{i})\right)(t-t_{k}{}^{i}), \end{array}$$

where *L*_*j*, ·_ represents *L*_*j, k*_, and *k* = 0, 1, 2, ⋯ , *N*.

According to the above expressions and the distributed event-triggering control function (11), we get

(37) $$\begin{array}{l} \;(||\eta_{i}(t_{k}{}^{i})(t-t_{k}{}^{i})+\varepsilon_{i}(t_{k}{}^{i})|{|}^{2}\text{+||}\\ \;(k\sum_{j=1}^{N}\left(L_{j,\cdot }(t_{k}{}^{i})+B_{j,\cdot }(t_{k}{}^{i}))\varepsilon_{j}(t_{k}{}^{i}\right)\\ -kr\sum_{j=1}^{N}\left(L_{j,\cdot }(t_{k}{}^{i})+B_{j,\cdot }(t_{k}{}^{i}\right))\eta_{j}(t_{k}{}^{i}))(t-t_{k}{}^{i})+\eta_{i}(t_{k}{}^{i}){\text{||}}^{2})\\ \;\leq \gamma (\text{||}-\eta_{i}(t_{k}{}^{i})(t-t_{k}{}^{i}){\text{||}}^{2}+||\\ \;-(-k\sum_{j=1}^{N}\left(L_{j,\cdot }(t_{k}{}^{i})+B_{j,\cdot }(t_{k}{}^{i}))\varepsilon_{j}(t_{k}{}^{i}\right)\\ \;-kr\sum_{j=1}^{N}\left(L_{j,\cdot }(t_{k}{}^{i})+B_{j,\cdot }(t_{k}{}^{i}))\eta_{j}(t_{k}{}^{i}))\right)(t-t_{k}{}^{i})|{|}^{2}). \end{array}$$

In order to simplify (37), we define

(38) $$\Omega \text{=}{\left\|\begin{array}{l} -(-k\sum_{j=1}^{N}\left(L_{j,\cdot }(t_{k}{}^{i})+B_{j,\cdot }(t_{k}{}^{i}))\varepsilon_{j}(t_{k}{}^{i}\right)\\ -kr\sum_{j=1}^{N}\left(L_{j,\cdot }(t_{k}{}^{i})+B_{j,\cdot }(t_{k}{}^{i}))\eta_{j}(t_{k}{}^{i})\right) \end{array}\right\|}^{2}+{\left\|-\eta_{i}(t_{k}{}^{i})\right\|}^{2},$$

and

(39) $$\begin{array}{l} \pi \text{=}-k(\sum_{j=1}^{N}\left(L_{j,\cdot }(t_{k}{}^{i})+B_{j,\cdot }(t_{k}{}^{i}))\varepsilon_{j}(t_{k}{}^{i}\right))\\ \;-kr(\sum_{j=1}^{N}\left(L_{j,\cdot }(t_{k}{}^{i})+B_{j,\cdot }(t_{k}{}^{i}\right))\eta_{j}(t_{k}{}^{i})). \end{array}$$

Suppose $\sigma_{i}\text{=}t-t_{k}^{i}$, we have

(40) $${\left\|\eta_{i}(t_{k}{}^{i})\sigma_{i}+\varepsilon_{i}(t_{k}{}^{i})\right\|}^{2}+{\left\|\pi \sigma_{i}+\eta_{i}(t_{k}{}^{i})\right\|}^{2}\leq \gamma \Omega \sigma_{i}^{2}.$$

We can see that when σ_*i*_ = 0, the inequality is not true. So σ_*i*_ > 0, that is to say $t-{t_{k}}^{i}>0$. To sum up, the self-triggering control strategy of the follower vehicle at $t_{k}^{i+1}$ moment is determined by the following conditions

(41) $${\left\|\eta_{i}(t_{k}{}^{i})\sigma_{i}+\varepsilon_{i}(t_{k}{}^{i})\right\|}^{2}+{\left\|\sigma_{i}+\eta_{i}(t_{k}{}^{i})\right\|}^{2}=\gamma \Omega \sigma_{i}^{2}.$$

σ_*i*_ > 0 which satisfies (41), we get the next trigger time $t_{k}^{i+1}=\sigma_{i}+t_{k}^{i}$. In particular, if the topology of the vehicle queue changes at time *t*, so that $t_{k}^{i+1}=t.$

**REMARK 2**. *The existence of σ_*i*_ indicates that a Zeno behavior does not exist. At the same time, it indicates that the self-triggered control strategy can realize the leader-follower consistency of vehicle formation under the condition of time-varying topology and the leader vehicle speed being the same. The proof of stability is same to event-triggered control, so we are omitted here*.

#### 3.2.2. The Speed of the Leading Vehicle Is Time Varying

In this part, we will transform the event-triggered control (23) into a self-triggered control strategy for the case that the vehicle speed of the leader is time varying.

We know that from the previous distributed event triggering control. $\left(||\varepsilon_{i}\left(t\right)|{|}^{2}+||\eta_{i}\left(t\right)|{|}^{2}\right)\leq \gamma \left(||e_{i}^{x}\left(t\right)|{|}^{2}+||e_{i}^{v}\left(t\right)|{|}^{2}\right)$ where $\gamma =\frac{ka\lambda_{\min}\left(H\left(t\right)\right)}{2\zeta }-\frac{1}{2a_{1}\zeta }$.

Using Taylor's formula, expand $\varepsilon_{i},\eta_{i},e_{i}^{x},e_{i}^{v}$ at $t_{k}^{i}$, we have

(42) $$\begin{array}{l} \varepsilon_{i}(t)=\;\eta_{i}(t_{k}{}^{i})(t-t_{k}{}^{i})+\varepsilon_{i}(t_{k}{}^{i}),\\ \eta_{i}(t)=\dot{\eta_{i}}(t_{k}{}^{i})(t-t_{k}{}^{i})+\eta_{i}(t_{k}{}^{i})\\ \;=(f(t,\varepsilon (t_{k}{}^{i}),\eta (t_{k}{}^{i}),e^{x}(t_{k}{}^{i}),e^{v}(t_{k}{}^{i}))\\ \;-k\sum_{j=1}^{N}\left(L_{j,\cdot }(t_{k}{}^{i})+B_{j,\cdot }(t_{k}{}^{i}))\varepsilon_{j}(t_{k}{}^{i}\right)\\ \;-kr\sum_{j=1}^{N}\left(L_{j,\cdot }(t_{k}{}^{i})+B_{j,\cdot }(t_{k}{}^{i}))\eta_{j}(t_{k}{}^{i})\right)(t-t_{k}{}^{i})+\eta_{i}(t_{k}{}^{i}) \end{array}$$

and

(43) $$\begin{array}{l} e_{i}{}^{x}=\;\varepsilon_{i}(t_{k}{}^{i})-\varepsilon_{i}(t)=-\eta_{i}(t_{k}{}^{i})(t-t_{k}{}^{i})\\ e_{i}{}^{v}=\;\eta_{i}(t_{k}{}^{i})-\eta_{i}(t)=-\dot{\eta_{i}}(t_{k}{}^{i})(t-t_{k}{}^{i})\\ \text{ =}-(f(t,\varepsilon (t_{k}{}^{i}),\eta (t_{k}{}^{i}),e^{x}(t_{k}{}^{i}),e^{v}(t_{k}{}^{i}))\\ \;-k\sum_{j=1}^{N}\left(L_{j,\cdot }(t_{k}{}^{i})+B_{j,\cdot }(t_{k}{}^{i}))\varepsilon_{j}(t_{k}{}^{i}\right)\\ \;-kr\sum_{j=1}^{N}\left(L_{j,\cdot }(t_{k}{}^{i})+B_{j,\cdot }(t_{k}{}^{i}))\eta_{j}(t_{k}{}^{i})\right)(t-t_{k}{}^{i}). \end{array}$$

According to the above two formulas and (23), we get

(44) $$\begin{array}{l} ({\left\|\eta_{i}(t_{k}{}^{i})(t-t_{k}{}^{i})+\varepsilon_{i}(t_{k}{}^{i})\right\|}^{2}\\ \text{+}{\left\|\begin{array}{l} \left(f(t,\varepsilon (t_{k}{}^{i}),\eta (t_{k}{}^{i}),e^{x}(t_{k}{}^{i}),e^{v}(t_{k}{}^{i}\right)\\ -k\sum_{j=1}^{N}\left(L_{j,\cdot }(t_{k}{}^{i})+B_{j,\cdot }(t_{k}{}^{i}))\varepsilon_{j}(t_{k}{}^{i}\right)\\ -kr\sum_{j=1}^{N}\left(L_{j,\cdot }(t_{k}{}^{i})+B_{j,\cdot }(t_{k}{}^{i}))\eta_{j}(t_{k}{}^{i})\right)(t-t_{k}{}^{i})\\ +\eta_{i}(t_{k}{}^{i}) \end{array}\right\|}^{2})\\ \leq \gamma ({\left\|-\eta_{i}(t_{k}{}^{i})(t-t_{k}{}^{i})\right\|}^{2}\\ +\|\begin{array}{l} \begin{array}{l} -(f(t,\varepsilon (t_{k}{}^{i}),\eta (t_{k}{}^{i}),e^{x}(t_{k}{}^{i}),e^{v}(t_{k}{}^{i})\\ -k\sum_{j=1}^{N}\left(L_{j,\cdot }(t_{k}{}^{i})+B_{j,\cdot }(t_{k}{}^{i}))\varepsilon_{j}(t_{k}{}^{i}\right)\\ -kr\sum_{j=1}^{N}\left(L_{j,\cdot }(t_{k}{}^{i})+B_{j,\cdot }(t_{k}{}^{i}))\eta_{j}(t_{k}{}^{i})\right)(t-t_{k}{}^{i}) \end{array} \end{array}{\|}^{2}). \end{array}$$

In order to simplify (44), we define

(45) $$\Omega \text{ = }\|\begin{array}{l} -(f(t,\varepsilon (t_{k}{}^{i}),\eta (t_{k}{}^{i}),e^{x}(t_{k}{}^{i}),e^{v}(t_{k}{}^{i})\\ -k\sum_{j=1}^{N}\left(L_{j,\cdot }(t_{k}{}^{i})+B_{j,\cdot }(t_{k}{}^{i}))\varepsilon_{j}(t_{k}{}^{i}\right)\\ -kr\sum_{j=1}^{N}\left(L_{j,\cdot }(t_{k}{}^{i})+B_{j,\cdot }(t_{k}{}^{i}))\eta_{j}(t_{k}{}^{i})\right) \end{array}{\|}^{2}+{\left\|-\eta_{i}(t_{k}{}^{i})\right\|}^{2},$$

(46) $$\begin{array}{l} \pi \text{ = }f(t,\varepsilon (t_{k}{}^{i}),\eta (t_{k}{}^{i}),e^{x}(t_{k}{}^{i}),e^{v}(t_{k}{}^{i})\\ \;-k\sum_{j=1}^{N}\left(L_{j,\cdot }(t_{k}{}^{i})+B_{j,\cdot }(t_{k}{}^{i}))\varepsilon_{j}(t_{k}{}^{i}\right)\\ \;-kr\sum_{j=1}^{N}\left(L_{j,\cdot }(t_{k}{}^{i})+B_{j,\cdot }(t_{k}{}^{i}))\eta_{j}(t_{k}{}^{i}\right). \end{array}$$

Suppose $\sigma_{i}\;\text{=}\;t-t_{k}^{i}$, we have

(47) $${\left\|\eta_{i}(t_{k}{}^{i})\sigma_{i}+\varepsilon_{i}(t_{k}{}^{i})\right\|}^{2}+{\left\|\pi \sigma_{i}+\eta_{i}(t_{k}{}^{i})\right\|}^{2}\leq \gamma \Omega \sigma_{i}^{2}.$$

We obtain that σ_*i*_ = 0, and the inequality is not true; σ_*i*_>0 that is to say $t-{t_{k}}^{i}>0$. To sum up, the self-triggering control strategy of the follower vehicle at $t_{k}^{i+1}$ moment is determined by the following conditions:

(48) $${\left\|\eta_{i}(t_{k}{}^{i})\sigma_{i}+\varepsilon_{i}(t_{k}{}^{i})\right\|}^{2}+{\left\|\pi \sigma_{i}+\eta_{i}(t_{k}{}^{i})\right\|}^{2}=\gamma \Omega \sigma_{i}^{2}.$$

If there is a σ_*i*_>0 which satisfies (48), we get the next trigger time $t_{k}^{i+1}=\sigma_{i}+t_{k}^{i}$. In particular, if the topology of the vehicle queue changes at time *t*, so that $t_{k}^{i+1}=t.$

**REMARK 3**. *The existence of σ_*i*_ indicates that a Zeno behavior does not exist. At the same time, it indicates that the self-triggered control strategy can achieve the leader-follower consistency of vehicle formation under the circumstance that both the topology structure and the leader vehicle speed are time varying. The proof of stability is the same to the event-triggered control. It is thus avoided here*.

## 4. Simulation

In this section, we will give two numerical experiments to verify the correctness and validity of the above theorems. Both experiments are based on a leader-follower vehicle formation system, which consist of a leader vehicle and four follower vehicles.

Firstly, we verify that the speed of the leader vehicle is constant. The dynamic equation of leader and follower are shown below:

(49) $$\{\begin{array}{l} {\dot{x}}_{0}(t)=v_{0}(t),\\ {\dot{v}}_{0}(t)=0, \end{array}\{\begin{array}{l} {\dot{x}}_{i}(t)=v_{i}(t),\\ {\dot{v}}_{i}(t)=u_{i}(t), \end{array}$$

where *u*_*i*_(*t*) is defined in (5), *k* = 3.4, *r* = 1.2. and the parameters satisfy the conditions in Theorem 3.1.

**Figure 2:**
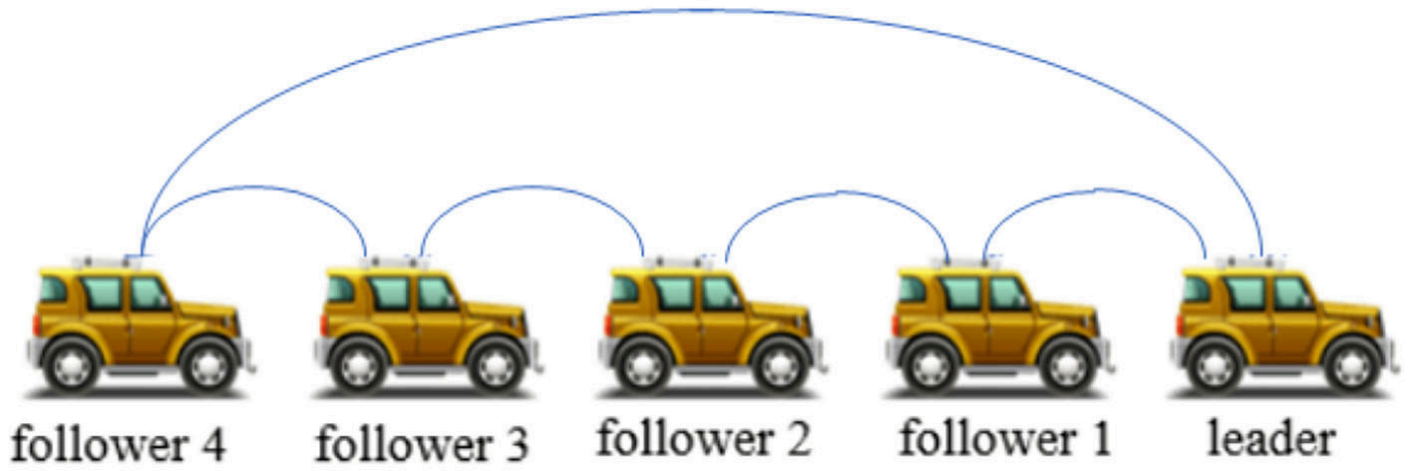
Topology at initial time.

In order to more intuitively verify the effectiveness of the self-triggering control strategy proposed in this paper, we assume that the vehicle formation system carries out three topology switches. The topology structure between vehicles at the initial moment is shown in Figure 2. Each adjacency matrix *A* and coefficient matrix *H* are defined as follows

$$\begin{array}{l} A_{1}=\left[\begin{array}{cccc} 0 & 1 & 0 & 0\\ 1 & 0 & 1 & 0\\ 0 & 1 & 0 & 1\\ 0 & 0 & 1 & 0 \end{array}\right],\;A_{2}=\left[\begin{array}{cccc} 0 & 1 & 0 & 1\\ 1 & 0 & 0 & 0\\ 0 & 0 & 0 & 1\\ 1 & 0 & 1 & 0 \end{array}\right],\\ A_{3}=\left[\begin{array}{cccc} 0 & 1 & 1 & 0\\ 1 & 0 & 0 & 0\\ 1 & 0 & 0 & 1\\ 0 & 0 & 1 & 0 \end{array}\right],H_{1}=\left[\begin{array}{cccc} 0 & -0.1 & -0.2 & -0.3\\ 0.1 & 0 & -0.1 & -0.2\\ 0.2 & 0.1 & 0 & 0.1\\ 0.3 & 0.2 & 0.1 & 0 \end{array}\right],\\ \;H_{2}=\left[\begin{array}{cccc} 0 & -0.1 & -0.2 & 0.1\\ 0.1 & 0 & -0.1 & 0.2\\ 0.2 & 0.1 & 0 & 0.3\\ -0.1 & -0.2 & -0.3 & 0 \end{array}\right],\\ \;H_{3}=\left[\begin{array}{cccc} 0 & 0.3 & 0.2 & 0.1\\ -0.3 & 0 & 0.1 & -0.2\\ -0.2 & 0.1 & 0 & -0.1\\ -0.1 & 0.2 & 0.1 & 0 \end{array}\right]. \end{array}$$

The initial values of the leader vehicle and the follower vehicle are defined as follows:

(50) $$\begin{array}{l} x_{0}(0)=(0,0),\;x_{1}(0)=(-0.4,-0.5),\;x_{2}(0)=(-0.3,-0.3),\\ x_{3}(0)=(-0.2,-0.4),x_{4}(0)=(-0.2,-0.1),\\ v_{0}(0)=(0.1,0.1),\;v_{1}(0)=(0.15,0.1)\\ v_{2}(0)=(0.1,0.12),\\ v_{3}(0)=(0.15,0.1),\;v_{4}(0)=(0.18,0.2). \end{array}$$

**Figure 3:**
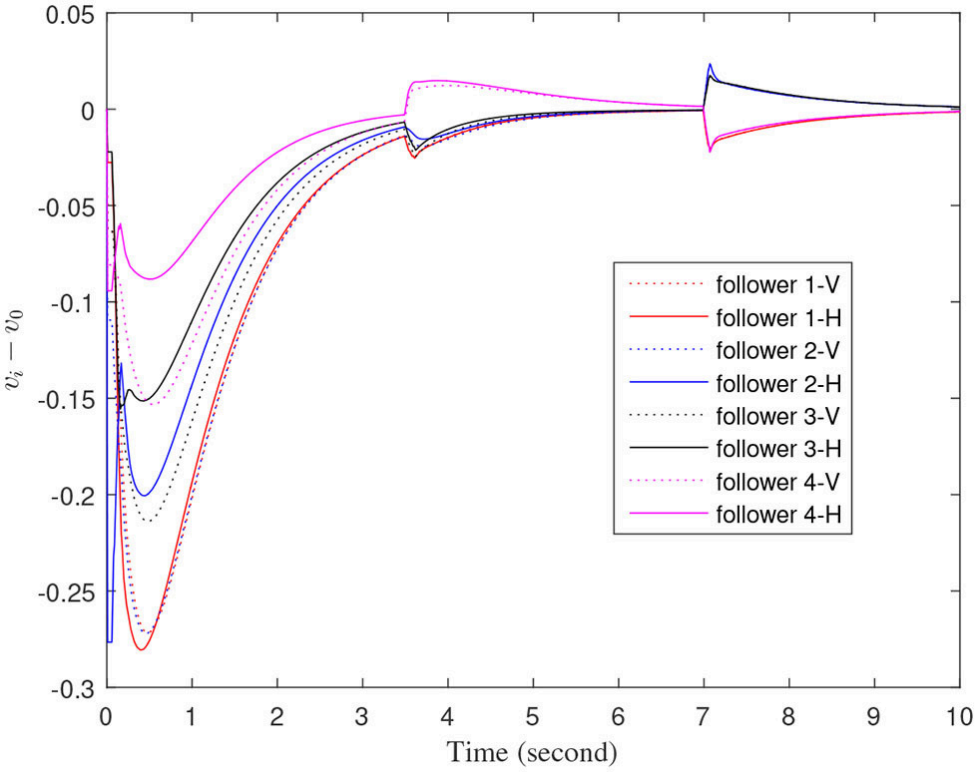
The velocity error between the follower car and the leader car.

**Figure 4:**
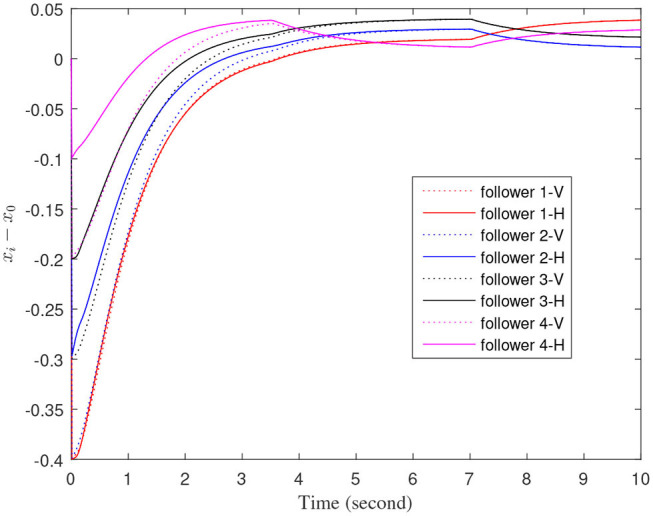
The real-time distance between each follower car and the leader car.

**Figure 5:**
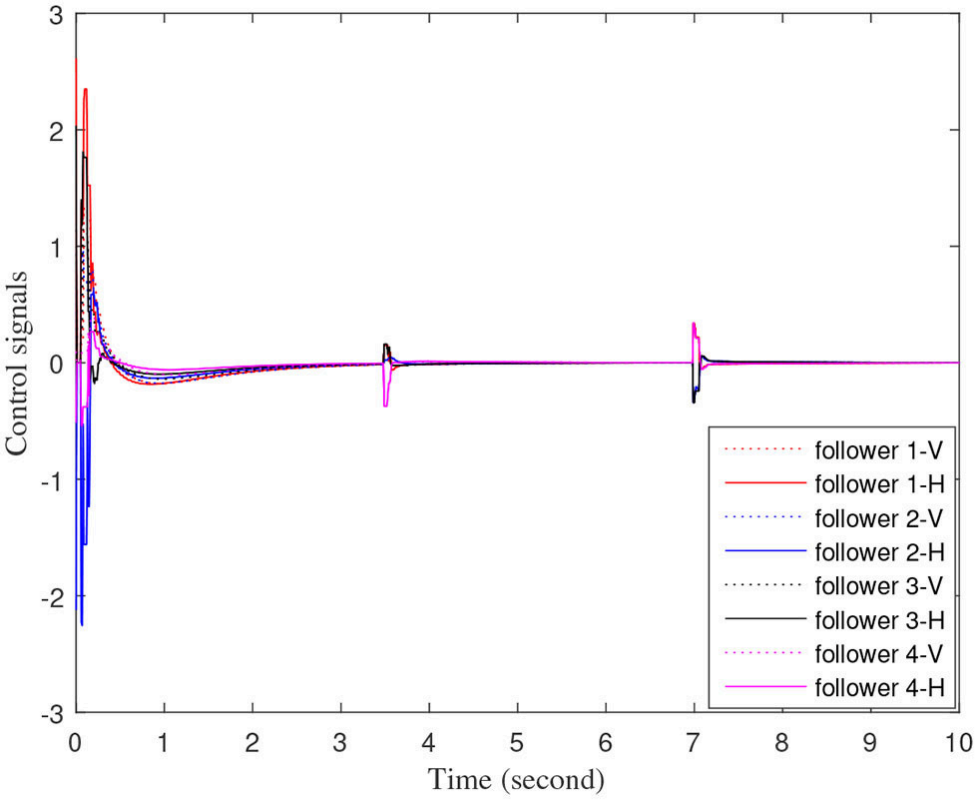
Control input signals of each follower vehicle.

**Figure 6:**
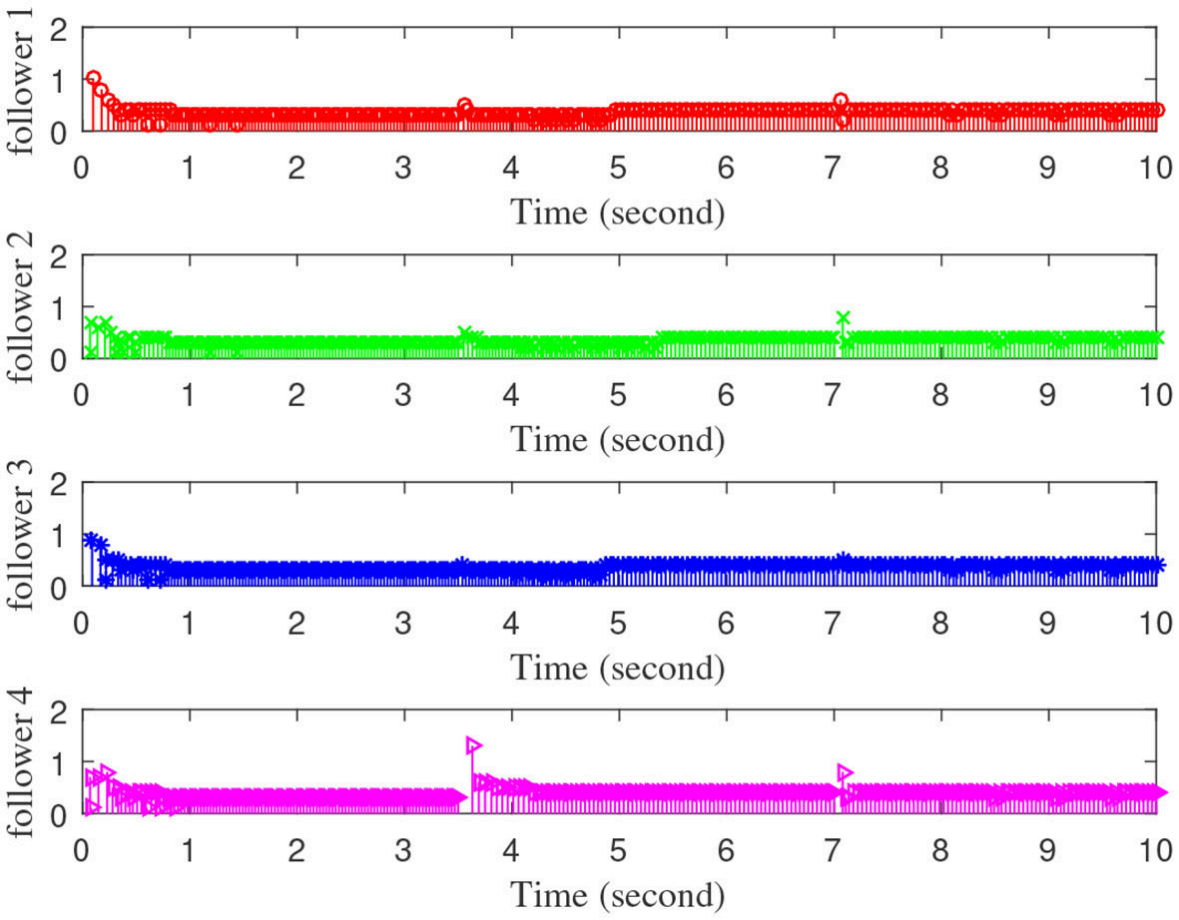
The event trigger interval of each follower vehicle.

**Figure 7:**
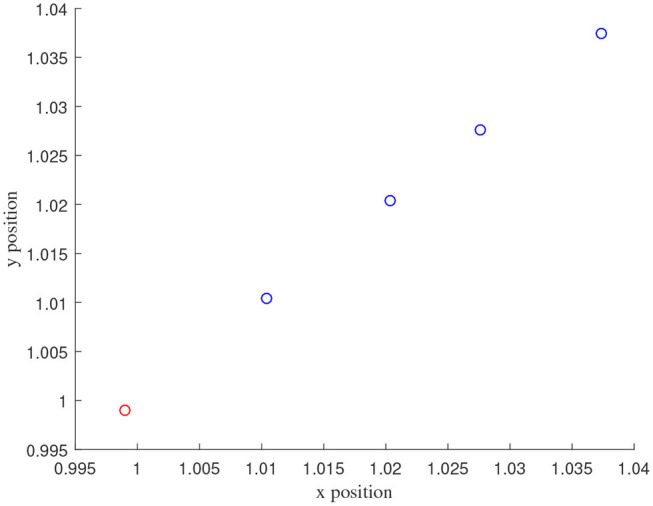
The position information of the vehicle when the formation is stable.

Figures 3–7 are the results for the leader vehicle at constant speed. Figure 3 shows the velocity error between the follower car and the leader car. Figure 4 express as the real-time distance between each follower car and the leader car. Figure 5 express as the changes in the controller of each follower car. Because the topology is changed, the controller changed dramatically twice. The self-trigger interval of each follower are displayed in Figure 6. Figure 7 shows the relative position of vehicles when the formation is finally stabilized.

As we can see from Figures 3–5, when the topology changes, the controller of the follower vehicle adjusts the vehicle speed to keep the vehicle in formation and the error of vehicle speed tends to zero over time. This indicates that the controller can adapt to various topological switching situations by adjusting the control intensity. At the same time, due to the change of topology, the relative positions between vehicles will also change, and the follower vehicles will constantly adjust their positions to the new relative positions under the action of the controller. It is worth noting that the position error of the follower vehicles does not gradually approach zero as time goes on, and it reaches a fixed value greater than zero in Figure 4. This fixed value is the safe distance (*h*_*ij*_*v*_0_) between the vehicles. As shown in Figure 7, when a stable state is reached, the vehicles should keep a safe distance from each other. Moreover the self-triggering instants are displayed in Figure 6. The simulation results exhibit that the controller and the self-triggering control strategy designed by us have a good performance. It achieves the stability of vehicle formation system under the condition of constant topological changes. Moreover, the vehicles can keep a safe distance.

Secondly, we verify that the speed of the leader vehicle is time varying. In order to more intuitively verify the effectiveness of the self-triggering control strategy, we randomly selected several time points and used the leader vehicle dynamics Equation (4) to change the state of the leader vehicle. The dynamic equation of the leading vehicle are as follows

(51) $$\{\begin{array}{l} {\dot{x}}_{0}(t)={\dot{v}}_{0}(t),\\ {\dot{v}}_{0}(t)=-\sin\left(x_{0}(t)\right)-0.25v_{0}(t)+1.5\;\cos(2.5t). \end{array}$$

When the speed of leader changes, the dynamic equation of the follower's vehicle is defined as

(52) $$\{\begin{array}{l} {\dot{x}}_{i}(t)={\dot{v}}_{i}(t),\\ {\dot{v}}_{i}(t)=f\left(t,x_{i},v_{i}\right)-f(t,x_{0},v_{0})+u_{i}(t), \end{array}$$

where *f*(*t, x, v*) = −sin(*x*)−0.25*v*+1.5cos(2.5*t*).

**Figure 8:**
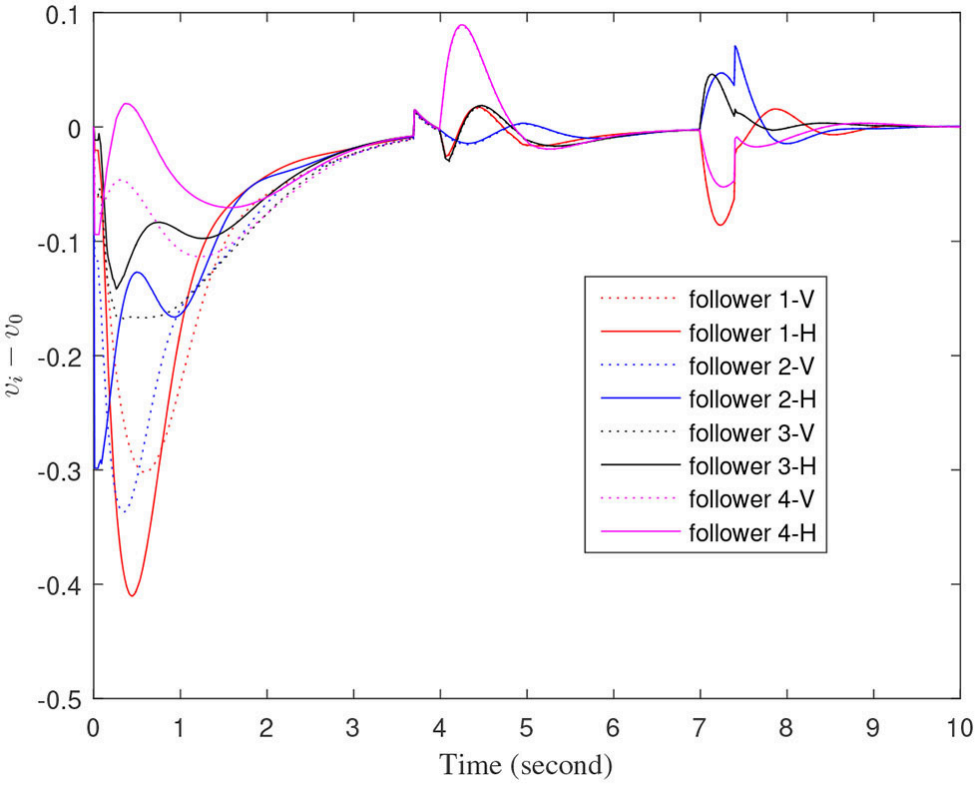
The velocity error between the follower car and the leader car.

**Figure 9:**
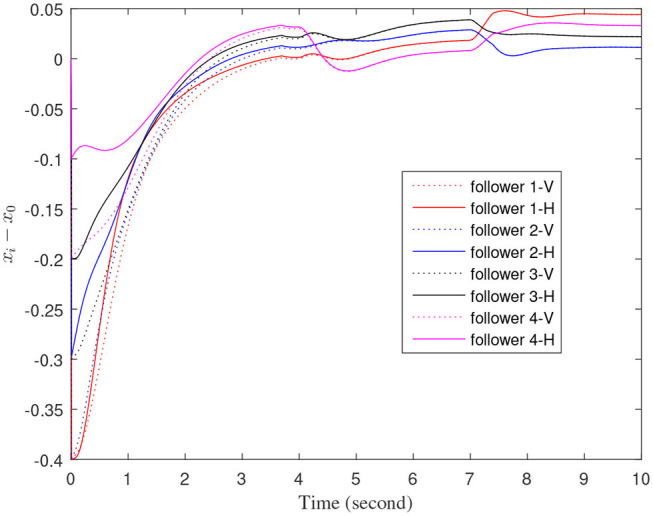
The real-time distance between each follower car and the leader car.

**Figure 10:**
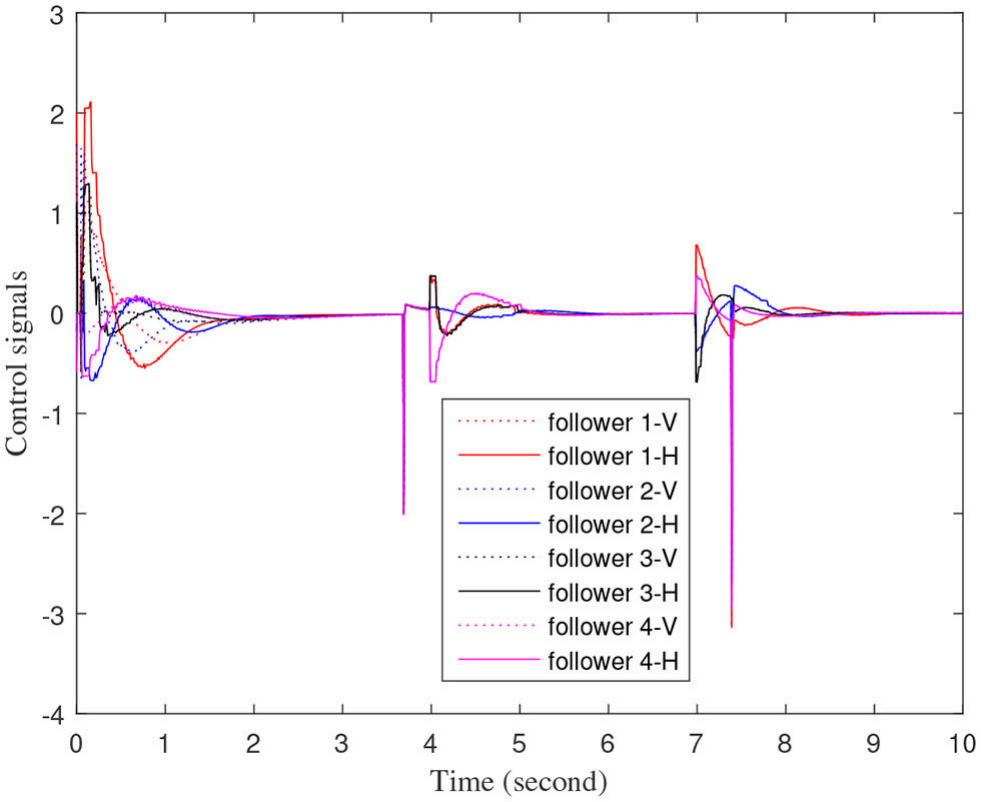
Control input signals of each follower vehicle.

**Figure 11:**
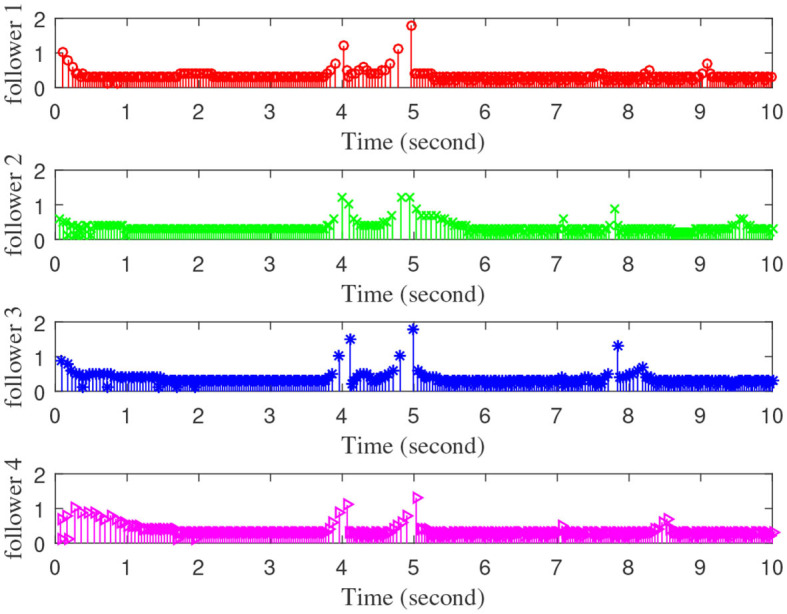
The event trigger interval of each follower vehicle.

**Figure 12:**
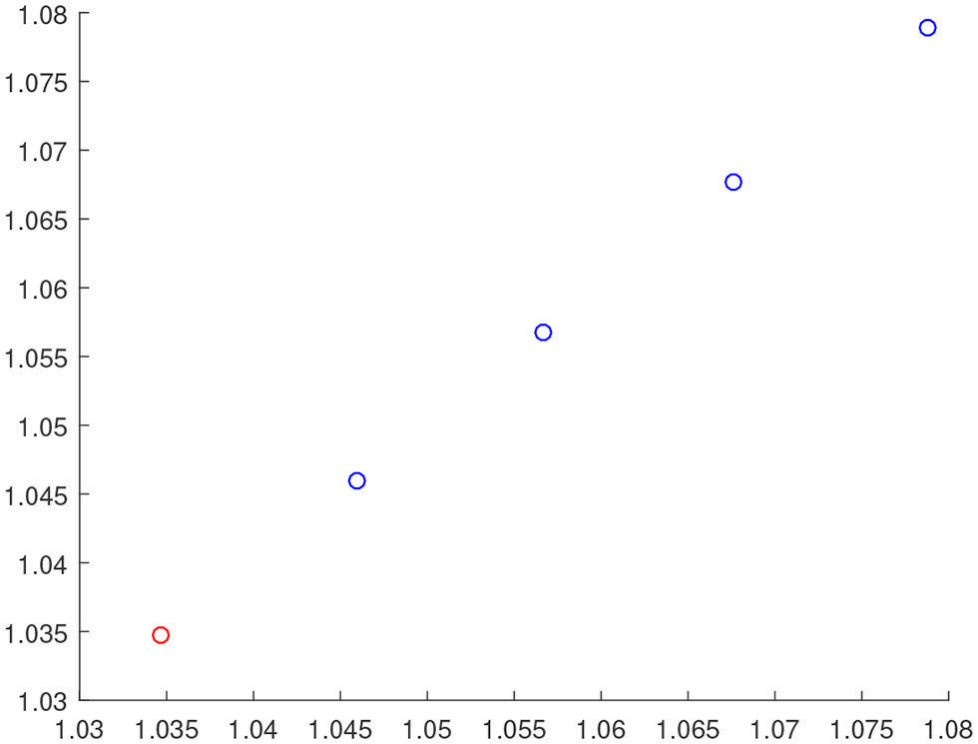
The position information of the vehicle when the formation is stable.

The define of Figures 8–12 is similar to Figures 3–7, but Figures 8–12 show the results of a leader with time-varying velocity. From Figures 8–10, we can see that when the speed of the leader vehicle or topology changes, the follower vehicle can quickly adapt to the changing so that its speed is consistent with the leader vehicle, and the real-time distance between follower vehicle and leader vehicle change rapidly. Moreover, it can be seen from Figure 10, that after the vehicle formation system reaches stability, the controller of the follower vehicle no longer exerts control. The self-triggering instants are displayed in Figure 11. Notably, after the leader vehicle speed changes, the safety distance of the follower vehicle also changes in Figure 9. However, the vehicles ultimately kept a safe distance, as shown in Figure 12. The simulation results show that the controller and the self-triggering control strategy designed in this paper have a good performance. It can make the vehicle formation system reach stable state under the condition of changing topology and leader speed.

The number of triggers with a distributed event-triggered control scheme in Yang et al. (2018) and self-triggered control scheme (41) within 0–15 s are shown in the Table 1. What we can obtain from Table 1 is that the self-triggered control scheme (41) needs less triggering events than the distributed event-triggered control scheme in Yang et al. (2018). At the same time, the mean time interval which represents the average time between each trigger in Table 2 indicates that the self-triggered control strategy designed in this paper has a lower trigger probability and execution moment. It shows that the self-triggered control strategy proposed here can effectively reduce the energy loss of data detection and calculation in the control process.

**Table 1:** Triggered numbers of follower agents.

**Control strategy**	**Numbers of triggered events of agents**			
	**1**	**2**	**3**	**Total**
Event-triggered in Yang et al. (2018)	582	857	1,029	2,468
Self-triggered (41)	275	234	226	735

**Table 2:** Mean time interval of follower agents.

**Control strategy**	**Mean time interval**		
	**1**	**2**	**3**
Event-triggered in Yang et al. (2018)	0.0127	0.0119	0.0151
Self-triggered (41)	0.0546	0.0641	0.0662

## 5. Conclusions

In this paper, we have studied leader-follower consistency in vehicle formation systems with time-varying topology under event-triggering mechanism. The difference between our work and the published papers is that we have designed a self-triggering control strategy that avoids continuous calculation and measurement and reduces the loss of communication resources. At the same time, we have proved the consistency of the system under the control of the trigger function. In addition, we have also studied the consistency of the vehicle formation system with time-varying topology when the leader speed is time varying. Finally, the effectiveness of the proposed controllers has been verified by numerical experiments. In addition, it should be noted that, although we proved the stability of formation system by Lyapunov function, we did not give its string stability which will be studied in the future.

## Data Availability Statement

All datasets generated for this study are included in the article/supplementary material.

## Author Contributions

All authors listed have made a substantial, direct and intellectual contribution to the work, and approved it for publication.

## Conflict of Interest

The authors declare that the research was conducted in the absence of any commercial or financial relationships that could be construed as a potential conflict of interest.
